# Asymptomatic Simple Bone Cysts Revealed by Imaging: A Report of Two Cases

**DOI:** 10.7759/cureus.87166

**Published:** 2025-07-02

**Authors:** Sara Boukssim, Hajar Soualem, Bassima Chami

**Affiliations:** 1 Odontology Department, Mohammed V Military Training Hospital Mohammed V University in Rabat, Rabat, MAR

**Keywords:** case report, intraosseous lesion, oral cavity, radiolucency, simple bone cyst

## Abstract

Simple bone cysts (SBCs) are rare benign intraosseous lesions typically discovered incidentally during radiographic examinations. This report presents two cases of mandibular SBCs in young North African males. The first case involved a 15-year-old with an asymptomatic radiolucent lesion between teeth 44 and 45, incidentally found after dental trauma. The second case concerned a 22-year-old with a swelling and a separate asymptomatic radiolucent lesion extending from tooth 34 to 38. In both cases, imaging revealed well-defined unilocular lesions with scalloped borders and no cortical expansion. Surgical exploration uncovered empty bone cavities containing minimal serosanguinous fluid. Histopathological analysis confirmed the diagnosis of SBCs by identifying fibrous connective tissue lacking epithelial lining. Both patients underwent successful surgical curettage. These cases underscore the importance of radiographic, clinical, and histological correlation in diagnosing SBCs and highlight surgical exploration as both diagnostic and therapeutic. Accurate recognition is essential to avoid unnecessary aggressive treatment.

## Introduction

Simple bone cysts (SBCs), also known as traumatic bone cysts, are benign intraosseous lesions characterized by empty or fluid-filled cavities without an epithelial lining. Typically, asymptomatic and discovered incidentally, these cysts predominantly affect young patients and frequently involve the mandible [1]. Despite their benign nature, accurate diagnosis and differentiation from other odontogenic and non-odontogenic lesions are crucial due to overlapping radiographic features. Herein, we describe two clinical cases of an incidental mandibular simple bone cyst identified during routine radiographic assessment.

## Case presentation

Case 1

A 15-year-old North African male was referred to the Department of Odontology at the Military Training Hospital following the incidental discovery of a radiolucent lesion during routine radiographic evaluation. The initial consultation was prompted by a recent traumatic episode resulting in an enamel-dentin fracture of tooth 21. During the radiological assessment, an unexpected radiolucency was noted in the mandibular right quadrant, between teeth 44 and 45.

**Figure 1:**
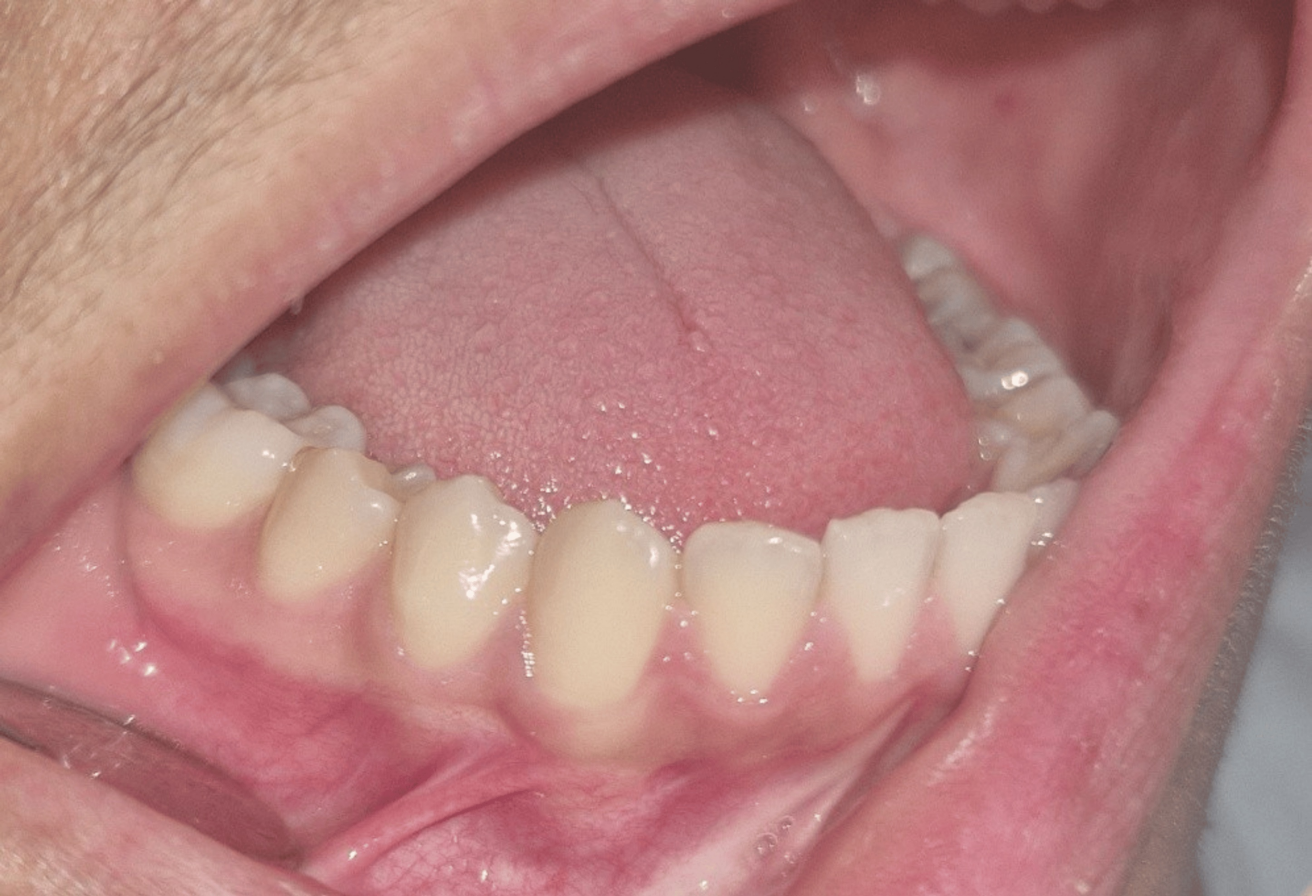
Intraoral view revealing no swelling in the region of teeth 44 and 45

The patient’s medical history was unremarkable, with no systemic conditions or prior dental pathologies reported, aside from the recent trauma. Extraoral examinations revealed no facial asymmetry or palpable abnormalities. Intraoral inspection of the region between teeth 44 and 45 showed no signs of swelling or mucosal changes. Teeth 44 and 45 were clinically healthy, responding positively to sensibility pulp testing and demonstrating no mobility or sensitivity to percussion (Figure 1).

**Figure 2:**
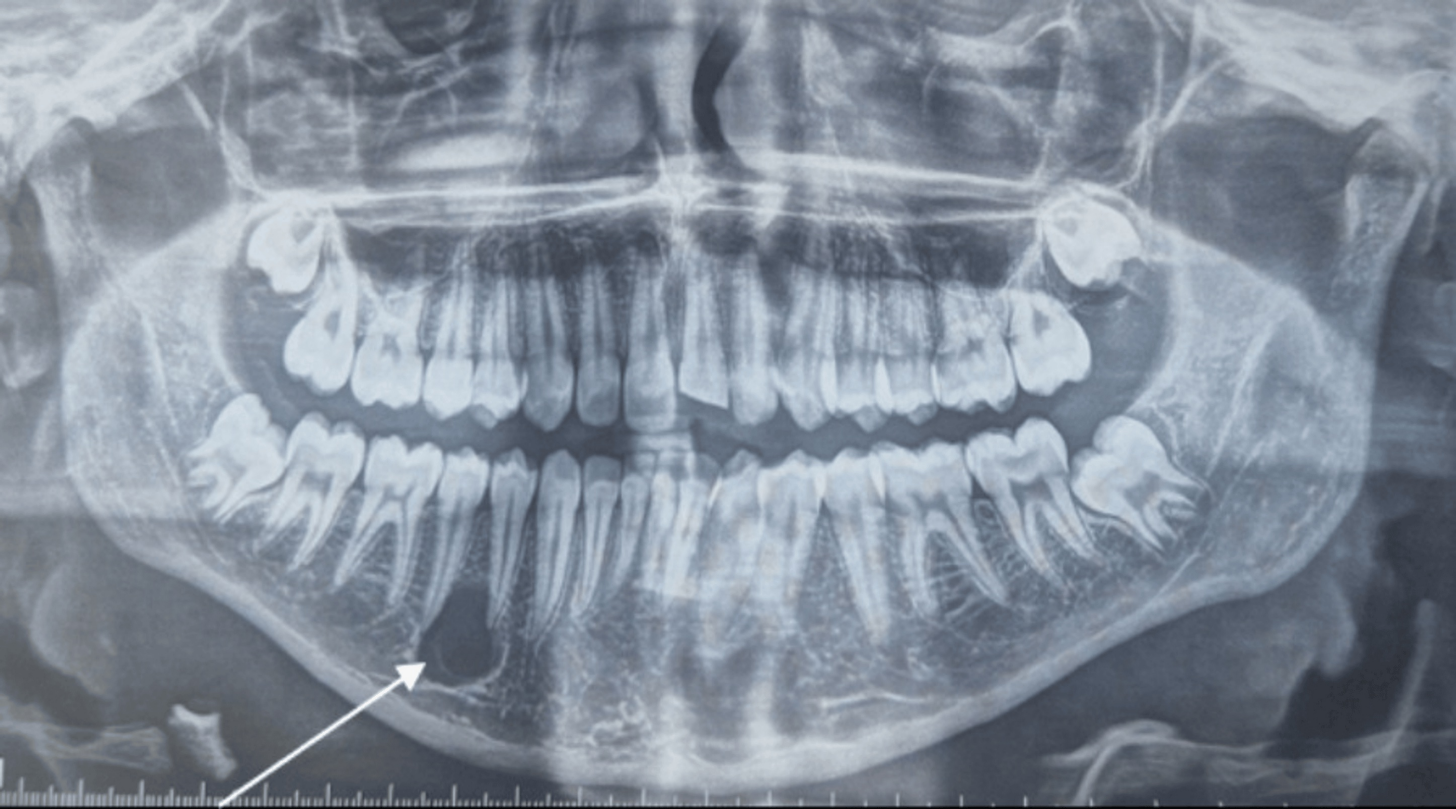
Orthopantomogram revealing a unilocular radiolucent image between the roots of teeth 44 and 45

The orthopantomogram exam revealed a well-defined, unilocular radiolucency with scalloped borders extending between the roots of teeth 44 and 45 (Figure 2).

Further imaging using cone-beam computed tomography (CBCT) confirmed a clearly demarcated hypodense lesion with no evidence of cortical expansion. The lesion was observed to extend near the mental foramen without causing displacement or invasion.

**Figure 3:**
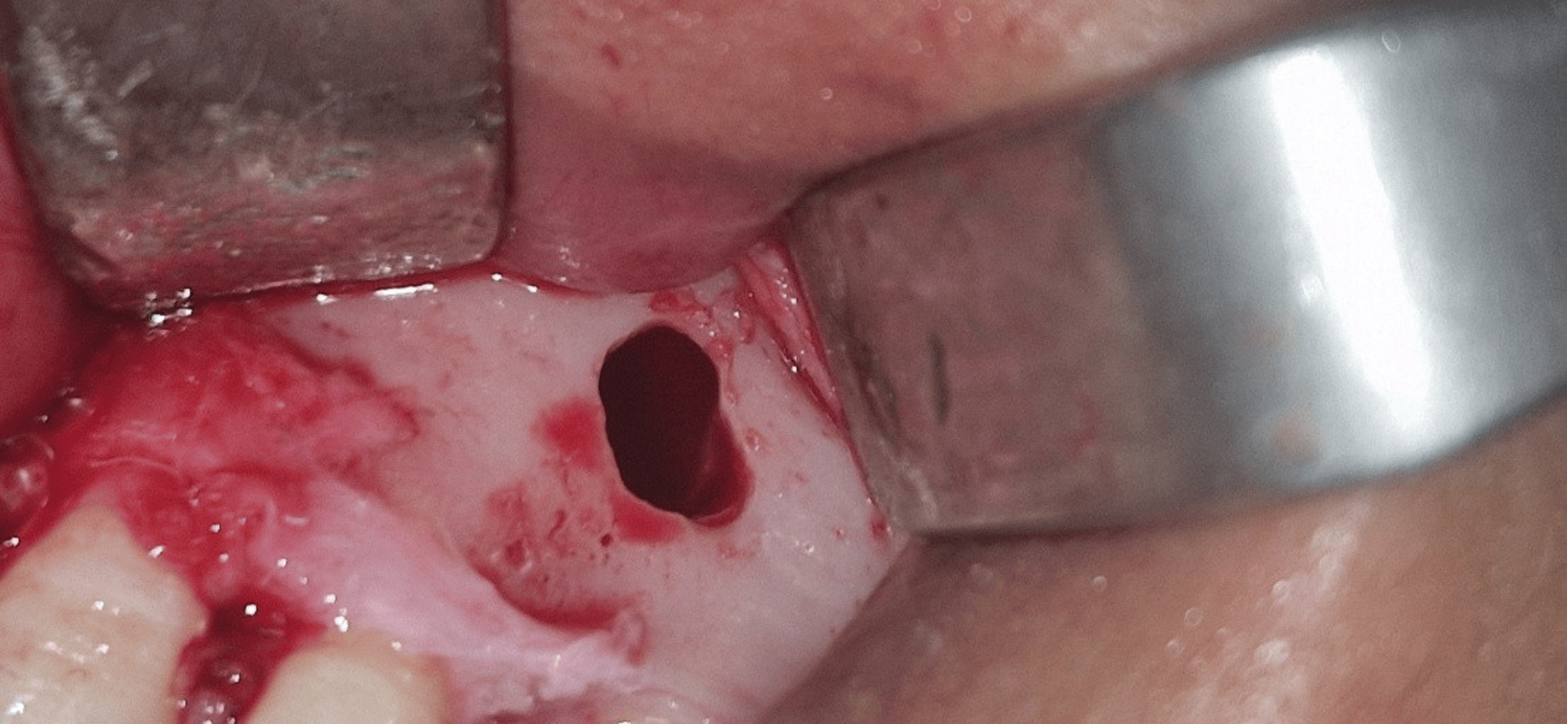
Surgical exploration revealed an empty bone cavity with smooth, shiny internal surfaces containing minimal serosanguinous fluid

Given the nonspecific radiological features, which overlapped with those of several odontogenic and non-odontogenic entities, surgical exploration was undertaken. Intraoperatively, an empty bone cavity was encountered, characterized by smooth, glistening internal walls and containing a small amount of serosanguinous fluid (Figure 3).

Histopathological examination of the cavity lining revealed a thin layer of vascularized fibrous connective tissue devoid of epithelial lining, establishing the definitive diagnosis of an SBC.

Case 2

**Figure 4:**
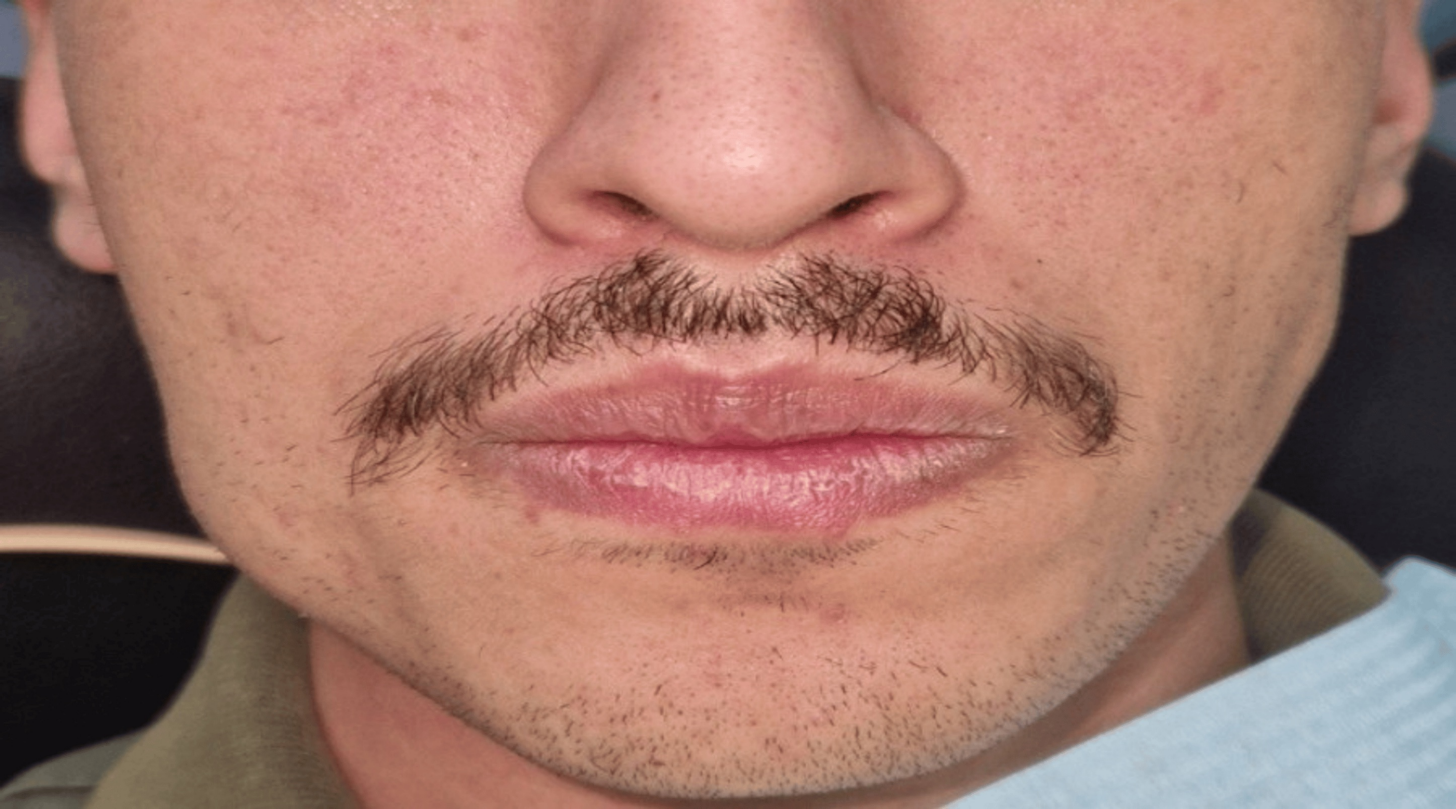
Extraoral view revealing a swelling in the posterior region of the right mandible

A 22-year-old North African male was referred to the Department of Odontology at the Military Training Hospital for evaluation of a swelling in the posterior region of the right mandible, which had been present for approximately three months (Figure 4).

During history taking, the patient reported a history of childhood trauma to the chin. The extraoral examination revealed a solitary, non-fluctuating swelling in the right side of the mandible. On intraoral examination, a solitary, non-tender, non-fluctuating circumferential swelling attached to the right cheek, located opposite to the second premolar and first molar, was noticed.

Additionally, an asymptomatic swelling was observed on the contralateral side of the mandible, extending from tooth 34 to tooth 38. The overlying mucosa appeared normal, and there were no signs of dental caries or other visible pathologies. The involved teeth were responsive to sensibility pulp testing, with no evidence of mobility or percussion sensitivity.

**Figure 5:**
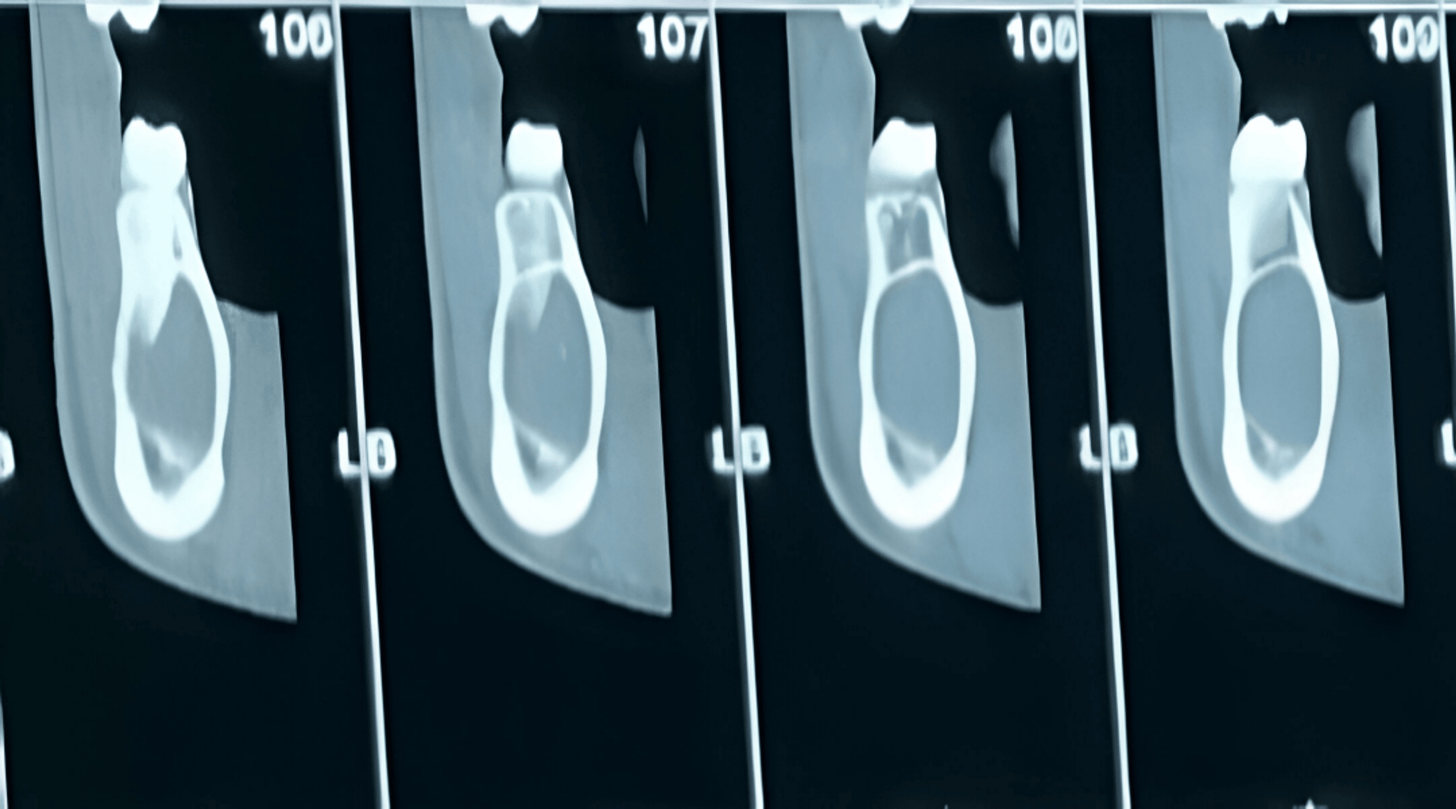
CBCT view revealing a unilocular lesion with preservation of the buccal and lingual cortices and displacement of the inferior alveolar nerve

CBCT further confirmed a well-demarcated lesion with low attenuation, showing no evidence of cortical expansion. The lesion was noted to approach the inferior alveolar nerve canal, causing its displacement (Figure 5).

Given the nonspecific radiographic features, which could potentially mimic various odontogenic and non-odontogenic lesions, surgical exploration and histopathological examination were deemed necessary for a definitive diagnosis. Surgical exploration revealed an empty bone cavity with smooth, shiny internal walls, containing a minimal quantity of serosanguinous fluid.

Histological examination of the cavity walls demonstrated a thin layer of vascular fibrous connective tissue without any epithelial lining, confirming the diagnosis of an SBC.

As for the lesion on the right buccal mucosa, further evaluation confirmed the diagnosis of lipoma.

This case report has been reported in line with the SCARE criteria [2].

## Discussion

SBCs represent intraosseous lesions characterized by cavities without epithelial cyst linings. Although traditionally described as empty spaces, SBCs can occasionally contain serous or serosanguinous fluid and may present a fibrous lining in certain cases [1,3].

This lesion has been described under various terminologies, including solitary bone cyst [4], hemorrhagic cyst [5], unicameral bone cyst [6], idiopathic bone cavity, and traumatic bone cyst [7]. Despite the frequent use of the term "traumatic bone cyst," the role of trauma in its pathogenesis remains uncertain, as many affected patients have no recollection of prior trauma, and the incidence of trauma in SBC patients is not demonstrably higher than in the general population.

Several theories have emerged to explain SBC pathogenesis, reflecting the limited understanding of this uncommon entity. The most widely accepted theory suggests that a traumatic event causes hemorrhage within the bone marrow, followed by a failure of the resulting hematoma to organize and be replaced by normal tissue. However, this hypothesis has been challenged by the frequent absence of trauma history among patients. Cohen [8] proposed an alternative mechanism involving disrupted interstitial fluid flow within the bone, suggesting that minor increases in fluid pressure could lead to cyst expansion. More recently, Mirra et al. [9] theorized that SBCs might represent intraosseous synovial cysts caused by developmental anomalies, where synovial tissue becomes entrapped within bone, continuing to secrete fluid. This model could account for the higher incidence observed in adolescents, coinciding with periods of significant skeletal growth and development [3].

Notably, both patients in the current report had a history of trauma, potentially supporting a traumatic origin in these cases.

Clinically, SBCs manifest equally in both genders, predominantly occurring around the age of 18 years, with a higher incidence reported in white individuals and typically affecting the posterior mandible [10]. In the present cases, both patients were of a young age.

SBCs are frequently discovered incidentally during routine radiographic evaluations, as was true in both reported cases. While SBS usually occurs as single lesions, it may also present as multiple lesions [3,11]. Patients generally remain asymptomatic, with occasional mild swelling or discomfort, despite normal overlying mucosa [1].

On radiographs, SBCs appear as well-defined, unilocular radiolucencies that often scallop between the roots of adjacent teeth. These teeth remain vital, with no signs of root resorption or displacement, features that help distinguish SBCs from more aggressive odontogenic lesions [12]. Nonetheless, radiographic similarity to other pathologies, such as radicular cysts or keratocystic odontogenic tumors, underscores the importance of accurate diagnosis to prevent inappropriate management.

Interestingly, Suei et al. [13] asserted that radiographic features of TBCs can be beneficial for forecasting the possible prognosis as well as for diagnosis and discovery of the lesion. According to the results of their study, lesions maintaining an intact lamina dura typically demonstrate spontaneous or post-treatment healing. Furthermore, the pattern of bony expansion, smooth or absent expansion versus nodular, can predict recurrence, with nodular expansions correlating to higher recurrence rates.

Histologically, the inner walls of the cavity are often lined by a thin layer of compressed connective tissue, which may show myxoid (gel-like) degeneration. Additionally, areas of immature, lace-like osteoid or spiky collagen fibers may be present along the cavity walls. Occasional multinucleated giant cells, macrophages, or hemosiderin deposits may also be seen, especially in lesions with prior hemorrhage [14].

An increasingly recognized, though rare, clinicopathological entity involves the co-occurrence of SBCs with cemento-osseous dysplasia (COD). Initially described in long bones by Jaffe [15] in 1958 and later histologically confirmed by Adler [16], this association was first reported in the jaws by Melrose et al. [17] in 1976, who found SBCs in 41% of their COD cases. Approximately 50 similar intraoral cases have since been reported [18], typically presenting in the mandible with a marked female predilection. Multiple lesions or those associated with osseous dysplasia exhibit increased recurrence potential [19].

Treatment options for SBCs vary, with surgical intervention commonly employed both diagnostically and therapeutically. Complete surgical resection or meticulous curettage combined with bone grafting is considered definitive, though incomplete curettage can predispose patients, particularly children, to recurrence [19,20]. Non-surgical management may be feasible in selected cases but necessitates rigorous long-term follow-up [21]. Surgery must be performed for symptomatic cases whenever boundaries change or size and cortical expansion occur [21].

Recent studies have evaluated alternative treatments such as steroid injections, reporting lower recurrence rates and reduced morbidity compared to surgical procedures, though repeated injections may be necessary [22].

In both cases presented here, complete surgical curettage of the cavity was performed, consistent with the standard of care for SBC management.

## Conclusions

Simple bone cysts, although benign and generally asymptomatic, can mimic more aggressive lesions radiographically. Accurate diagnosis relies on clinical, radiological, and histopathological correlation. Surgical exploration remains both diagnostic and therapeutic, confirming the diagnosis while often promoting spontaneous healing. Awareness and proper identification of SBCs are essential to avoid unnecessary extensive interventions and to ensure appropriate management and follow-up. In those cases, both patients underwent post-surgical follow-up, and healing was uneventful, with no signs of recurrence during the monitoring period.
